# Estimation of Hydration Degree of Blended Cements with the Help of k-Values

**DOI:** 10.3390/ma12152420

**Published:** 2019-07-29

**Authors:** Pavel Reiterman, Ondřej Holčapek, Vendula Davidová, Roman Jaskulski, Martin Keppert

**Affiliations:** 1 University Centre for Energy Efficient Buildings, Czech Technical University in Prague, Třinecká 1024, 273 43 Buštěhrad, Czech Republic; 2 Faculty of Civil Engineering, Czech Technical University in Prague, Thakurova 6, 166 29 Prague 6, Czech Republic; 3 Faculty of Civil Engineering, Mechanics and Petrochemistry, Warsaw University of Technology, Lukasiewicza St. 17, 09-400 Plock, Poland

**Keywords:** supplementary cementitious materials, fly ash, blast furnace slag, ceramic powder, degree of hydration, thermogravimetry

## Abstract

The growing utilization of various mineral additives in the building industry has caused concern worldwide to reduce the emissions of carbon dioxide from Portland cement (OPC) production. The present paper is focused on the determination of the degree of hydration of blended binding systems based on Portland cement. Blast furnace slag, fly ash, and ceramic powder are used in the study; they are applied by 12.5 wt.% up to 50% of OPC replacement. The evolution of the hydration process is monitored using thermogravimetry in selected time intervals to determine the degree of hydration; its ultimate value is obtained from numerical estimation using the Michaelis-Menten equation. However, due to the application of active mineral additives, the correction in terms of equivalent binder is conducted. Corrected values of the degree of hydration exhibit good fit with compressive strength.

## 1. Introduction

Global warming and consequent climate change have become crucial problems worldwide. They are the consequence of carbon dioxide emissions related to human industrial and agricultural activities. That is why the development of new environmentally friendly technologies is one of the main focuses of the scientific community, in all technical disciplines.

The building industry is one of the most polluting industries, in which cement production is responsible for approximately 5% of global CO_2_ emissions [1,2]. The progress achieved in cement processing (more efficient milling, alternative fuels) is mainly being focussed on, since it could substitute a significant part of cement clinker without the loss of required properties (supplementary cementitious materials, SCM) [2]. The SCM, or mineral additives, are successfully applied for periods of long duration, because they positively contribute to themechanical and predominantly long-term durability performance of concrete. Their positive environmental impact is powered by the fact that the most frequently used ones, such as fly ash, and blast furnace slag, rank as industrial wastes or by-products [2,3].

Blast furnace slag (BFS) originates during pig iron production as a by-product, which has found wide utilization in the building industry in past years. The specific property of BFS, in comparison with other mineral additives, is the content of lime (CaO), which determines its latent hydraulic properties concurrently with glass phases. However, for its practical use in the cement industry, its sudden cooling during iron production is necessary to ensure its stability [4]. The level of utilization of BFS well corresponds with the cement industry and valid standard EN 197-1 [5], which defines individual cement types, where CEM IV could contain up to 95% of BFS. This material is also often applied as an OPC replacement to improve selected technological properties, such as shrinkage reduction [6], reduction of hydration heat [4], and enhancement of durability [2]. A positive impact on the hydration processes in presence of BFS allows for the self-healing ability of concrete [7]; however, relatively high levels of replacement are necessary (over 50%). The beneficial impact of BFS application on the environment is well known and supported by a number of studies [3,8]; however, with respect to the actual situation in the market, a gradual lack of BFS is expected.

Fly ash (FA) is a by-product originating during electric energy production in coal-fired power plants. The quality of the coal, burning regime, and flue gas desulphurization technology significantly determines the quality of the fly ash [9,10,11]. The varying properties and composition of FA, especially non-burned carbon, has a negative effect on the air entraining of fresh mix. On the other hand, suitable fly ash contributes to increased porosity, whereas the pore system is formed by higher amount of smaller pores [12]; smooth particles of FA have a positive impact on the release of fine air bubbles [13,14]. Fly ash has become an important constituent of concrete technology, because it affects positively the number of engineering properties. It is often used in concrete mixes during the production of large structures [15,16], due to its positive impact on the evolution of hydration heat. Such concrete also tends to reduce cracking due to shrinkage, what positively affects final durability. This is why fly ash holds a significant position in self-compacting concrete (SCC) manufacture, where it can substitute up to 60% of OPC [12,17]; however, very high levels of replacement in the binary system limit such concrete for severe exposure classes, according to EN 206 [18]. On the other hand, advanced ternary systems, typically in presence of limestone filler (LF), exhibit increased durability [12,17,19]. 

Ceramic powder (CP) obtained from red clay ceramics was used in lime based composites in ancient times. Currently, its importance is rising due to the high amount of construction and demolition waste (C&D). The character of the ceramics offers a number of suitable applications in the field of solidification of heavy metals [20] or composites that are resistant to high temperatures [21,22]. However, its application as partial cement replacement lies is of primary scientific interest [23,24,25]. Its pozzolanic properties are determined by the process of firing, when the applied temperature (600–800 °C) causes the formation of reactive amorphous phases due to clay dehydroxylation [24,26]. CP contains approximately half the amount of reactive components in comparison with fly ash; however, the physical effect of its use allows one to formulate composite materials with high mechanical and durability performance [27,28].

The efficiency of SCM application is often described by using the pozzolanic strength activity index (PSAI) to describe the strength gain/loss for that replacement level [29,30,31]; however, mechanical properties are not convenient for the durability assessment. For this reason, the performance index (PI) was introduced, which allows for the comparison of a single studied property [32,33], including factors relating to durability. Nevertheless, the efficiency of mineral additives in technical praxis is included in the mix design through the *k*-value, which performs the contribution of a given additive in relation to OPC. According to EN 206 [18], *k*-value is for blast furnace slag 0.6–0.9 and 0.2–0.4 for fly ash, respectively; however, its implementation in the calculation of water/cement ratio differs depending on the actual type of cement and applied SCM replacement used. Unfortunately, this valid mechanism based on the durability assessment according to w/c ratio is applicable for selected cement types for the replacement of up to 33.3% of cement, which partially blocks the further implementation of various SCM to concrete. The inappropriateness of this solution is well documented in work of Gruyaert et al. [34] and proved by practical experiences with the “XF” concrete classes. On the other hand, Ribeiro et al. documents [35] the deficiency of the *k*-value concept concerning the long-term carbonation depth.

It is very complicated to establish the exact reaction capacity of blended systems due to the obvious heterogeneity of cement and the additive used. Reaction kinetics could be easily monitored by using calorimetry, which well documents reaction kinetics on the basis of heat evolution at early ages. However, from a long-term point of view it is more suitable to apply the hydration degree (α), which is most frequently determined using thermogravimetry. In addition, this method allows for the monitoring of carbonation. However, the fundamental problem of hydration degree determination is the assumption of the ultimate reaction capacity, or chemically bound water. Because there are no specific values of ultimate chemically bounded water for single additives, this essential problem is also mentioned by Deboucha et al. [36], who verified the proposed model via the experimental results of compressive strength development. The hydration degree shows the exact expression of the hydration mechanism, which offers an effective formulation of mix composition, especially for blended systems [36,37].

The degree of hydration is conventionally used in applied research and technical praxis for the prediction of various engineering properties of cement-based composites. However, the factors described above complicate its estimation. This paper is focused on the estimation of the hydration degree, which includes design procedures determined by valid standards.

## 2. Materials and Methods 

**Figure 1:**
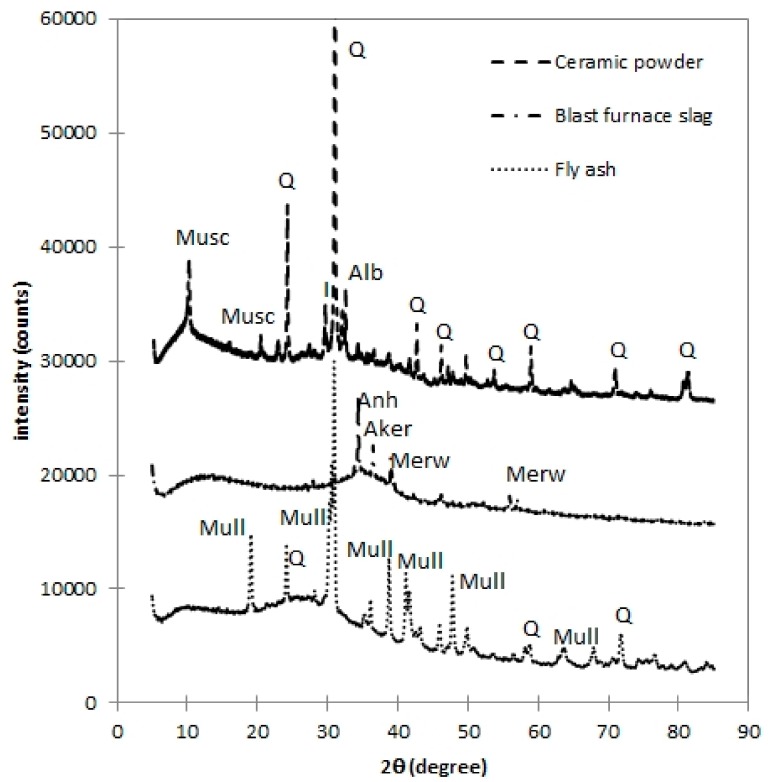
X-ray diffractograms of studied mineral additives (Q = quartz; Musc = muscovite; I = illite; Alb = albite; Anh = anhydrite; Aker = akermanite; Merw = merweinite; and Mull = mullite) [39].

**Figure 2:**
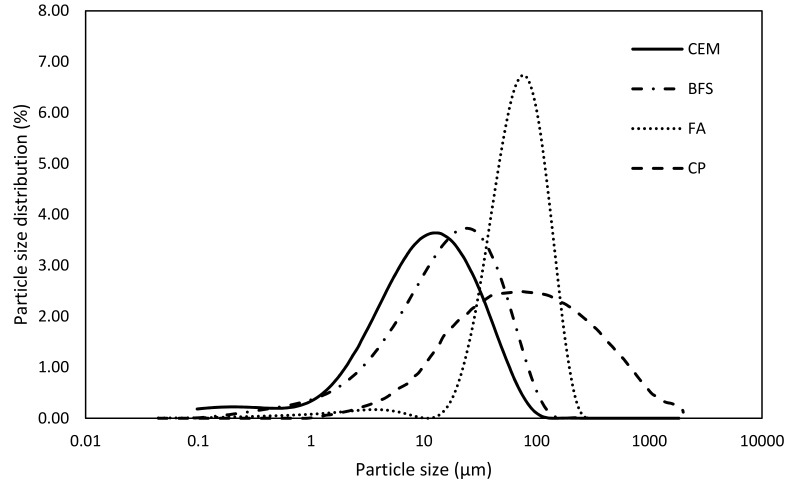
Particle size distribution of studied mineral additives.

The main aim of the experimental program was the determination of hydration degree using a combination of thermogravimetry and compressive strength measurement. Three various active additives were used in the paper—fly ash, blast furnace slag, and ceramic powder; their chemical composition determined by using device Thermo ARL 9400 XP (Waltham, MA, USA), and is introduced in Table 1. Mineral additives were applied, such as Portland cement CEM I 52.5 substitution. Applied replacement levels were 12.5, 25.0, 37.5, and 50.0 wt.%. Studied cement pastes were prepared with constant water to binder ratio of 0.55. Accompanying mortar mixtures were prepared with the above introduced binder systems and pure standard silica sand in ratio 1:4 (binder:sand) with similar w/b. The dosage of water was slightly increased, because studied Ceramic powder (CP) consumes part of mixing water due to its higher absorption; this corresponds to findings of Kannan et al. [28]. Despite different porosity along the series, the experiment structure and selection of constant w/b, respectively, reflects the commonly used approach in the technical praxis. All samples were cured under wet conditions to omit the carbonation of studied samples. The phase composition of the used mineral additives is introduced in Table 2. The XRD analysis of raw materials was performed by help of PAnalytical Aeris (Malvern Panalytical Ltd., Royston, UK) diffractometer equipped by Co_Kα_ tube operating at 40 kV and 7.5 mA. The incident beam path consisted of beta-filter iron, Soller slits 0.04 rad and divergence slit 1/2°. The diffracted beam path was equipped with 9 mm anti-scatter slit and Soller slits 0.04 rad. The used detector was PIXcel1D-Medipix3 detector (Malvern Panalytical Ltd.) with active length 5.542°. The scan ranged from 5 to 85°, step size 0.0027°, and counting time 2.0325 s. Data were evaluated by Profex software (ver. 3.12.1) (Solothurn Switzerland). The amount of amorphous portion was determined with help of added internal standard (20% of ZnO). Obtained Rietveld plots are illustrated in Figure 1. The particle size distribution was performed using laser diffraction for all studied binding materials due to understanding the reactivity and filler effect; resulting particle size distribution is shown in Figure 2.

Thermogravimetry. Thermogravimetric analysis was carried out by approximately 50 mg of the grind powder by monitoring weight loss in the range 105–1000 °C after 1, 7, 28, and 90 days, respectively. The thermal analysis was conducted directly in the above-mentioned ages; thus, the hydration of the samples was not arrested. The DTA-TG measurement (Schimadzu DTG-60H, Kyoto, Japan) was carried out in air atmosphere, and the heating rate was 10 °C/min. The amount of chemically bound water (H) and calcium hydroxide (CH) were expressed as wt.% of the dry sample, according to Equations (1) and (2). The exact boundaries of decomposition were identified by help of the derivative curves (DTG) [40,41]. The amount of water bound in Calcium Silicate Hydrate (CSH) and Calcium Aluminate Hydrate (CAH) hydrates was calculated as difference between total bound water and water bound in portlandite (Equation (3)).

(1) $$H=\frac{w_{105}-w_{550}}{w_{550}},$$

(2) $$CH=\frac{w_{450}-w_{550}}{w_{550}}\times \frac{74}{18}\;*,$$

(3) $$H_{hyd}=H-H_{CH},$$

* 74/18 is the ratio of molar weight of decomposed Ca(OH)_2_ and water H_2_O.

Nevertheless, even though paste samples were cured under wet conditions, decomposition in the range of calcite takes place; it is caused by the partial carbonation and the presence of non-aqueous phases in the used additives. This is why the weight loss in the range of approximately 650–850 °C was determined for all studied additives, which was subtracted proportionally according to applied replacement from the obtained weight loss during pastes testing, see Equation (4). This procedure allowed one to determine the amount of carbonated Portlandite, which has to be included into the calculation of chemically bounded water (Equation (3)) where *w_a,c_*, *s* mean, respectively, weight loss of used additive in the range of calcite and factor assessing the used substitution. This step has crucial sense in case of application limestone filler and an additive with content of calcite. The final determination of chemically bounded water can be expressed according to Equation (5).

(4) $$CH_{carb}=\frac{\left(w_{650}-w_{850}\right)}{w_{550}}-s\cdot w_{a,c},$$

(5) $$H_{hyd}=H-H_{CH}-H_{CHcarb}.$$

**Figure 3:**
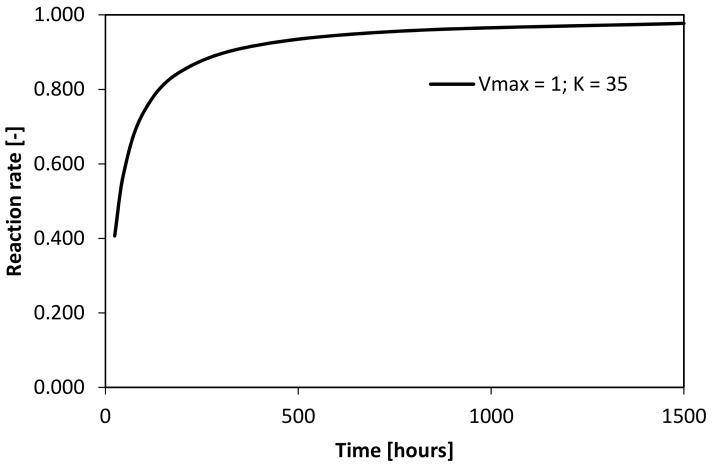
Illustration of Michaelis-Menten function.

Degree of hydration. Determination of degree of hydration (*α*) is derived from common equation (Equation (6)), which performs the ration of actual (*w_B_*) and ultimate amount of chemically bounded water (*w_B∞_*). In accordance with cement chemistry, ultimate value can be calculated on the basis of mineral composition of clinker. Bhatty et al. [37] applied the value 0.24, derived using Bogue’s formulas. However, it is necessary to estimate this crucial value. Michaelis-Menten equation seems to be suitable tool for the solution, because it well describes the reaction kinetics with given capacity; its common formulae is shown below (Equation (7)),

(6) $$\alpha =\frac{w_{B}}{W_{B\infty }},$$

(7) $$v=V_{max}\frac{T}{K+T},$$

where *V*_*max*_, *T*, K, and *v* mean ultimate reaction capacity, time (hours), constant (-), and degree of conversion, respectively. The illustration of this formulae for selected factors is shown in Figure 3. Although the cement-based binder hydration performs a complex of single reactions, the suitability of this approach was successfully applied by Deboucha et al. [36] and Monteagudo et al. [42]. Using that function, hydration mechanism could be easily approximated, where *V_max_* performs ultimate value of chemically bounded water and *v* content of hydrates in selected time (hours) determined by thermogravimetry. Estimation of the ultimate value was processed by Matlab using least square method (LSM), which offers the optimal fit of experimentally acquired data.

Equivalent binder mass. Effectiveness of various mineral additives could be evaluated in terms of compressive strength considering control mixture of OPC at selected age, usually 28 days—so called strength index (SI). This index, in fact, performs equivalent properties, when value 1 is equal to OPC. This means that the higher SI is the higher contribution to strength gain. This approach is partially adapted to k-value concept, when equivalent mass of binder is derived for the calculation of water-to-cement ratio (Equation (8)) in accordance to EN 206 [18], and k-value in fact performs conservative value of PSAI. There are introduced k-values for blast furnace slag, fly ash (class F), and silica fume in Table 3 applied in [36]. It is necessary to note that k-values could differ per a national standard.

(8) $$w/c=\frac{m_{w}}{L_{eq}}=\frac{m_{w}}{0.95\times m_{c}+k\cdot m_{a}},$$

*w/c*—water to cement ratio (-) prescribed for selected exposure classes;
*L_eq_*—equivalent mass of binder;
*m_w_*—dosage of water (kg);
*m_c_*—dosage of OPC (kg);
*k*—k-value (-);
*m_a_*—dosage of used additive (kg);
0.95—reflects content of clinker in the used Portland cement.

Compressive strength. Prismatic samples of dimensions 40 mm × 40 mm × 160 mm were used for the determination of compressive strength in accordance with EN 196-1 [43]. Compressive strength was determined on the fragments of the original prisms. Measurement of compressive strength was conducted after 1, 7, 28, and 90 days of curing under wet conditions using the set of three samples.

## 3. Results and Discussion

Performed experimental program was focused on the monitoring of the hydration process of blended binder systems on the basis of Portland cement, which was gradually replaced by the fly ash, blast furnace slag, and ceramic powder. Thermogravimetric measurements (product model, manufacturer, city, country) were used for the determination of the chemically bounded water in the hydrates. Final values of obtained chemically bounded water in hydrates (*H_hyd_*) are shown in following figures. The determination is described in Equations (1)–(5).

**Figure 4:**
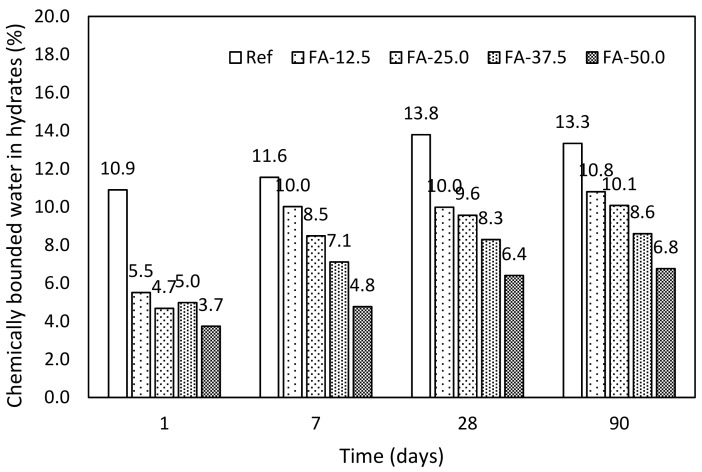
Chemically bounded water in hydrates—pastes with fly ash.

Pastes with addition of fly ash. There is introduced evolution of water bounded in hydrates in cement paste with addition of fly ash in the Figure 4, where the difference in the kinetics of hydration of pure cement paste and blended system, especially after 24 h, is obvious. On the other hand, during the first week there is evident gradual increase of the amount of hydrates. This is caused by the delayed hydration in presence of FA. The substitution by fly ash exceeding 25.0% exhibited very low progress of hydration, with final (90 days) amount of hydrates of 65% and 51%, respectively, compared to the control mixture. 

**Figure 5:**
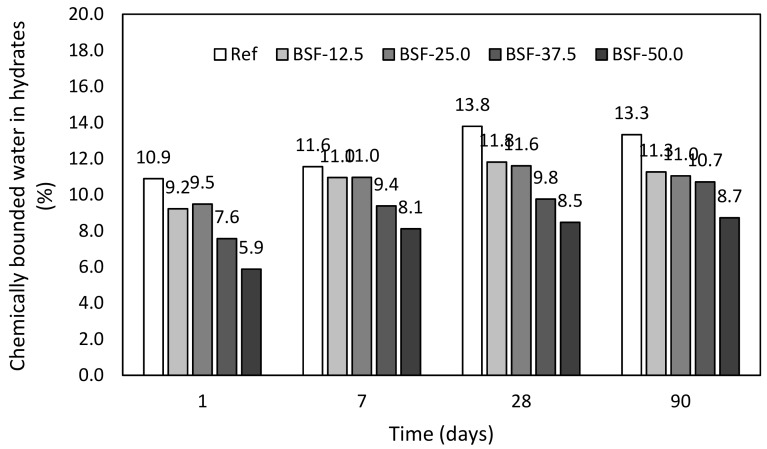
Chemically bounded water in hydrates—pastes with blast furnace slag.

Pastes with addition of blast furnace slag. Slightly different results were determined in case of paste with blast furnace slag, Figure 5. Initial amount of hydrates of paste incorporating BFS exhibit higher values in comparison with the FA. This is caused by the different substance of the blast furnace slag, which contains certain amount of lime, ensuring its latent hydraulic activity. This essential property allows considerably higher substitution for practical application; it is visible in the amount of hydrates of paste with the replacement by 12.5% and 25.0%, respectively, which performs the most frequently used substitutions in concrete technology.

**Figure 6:**
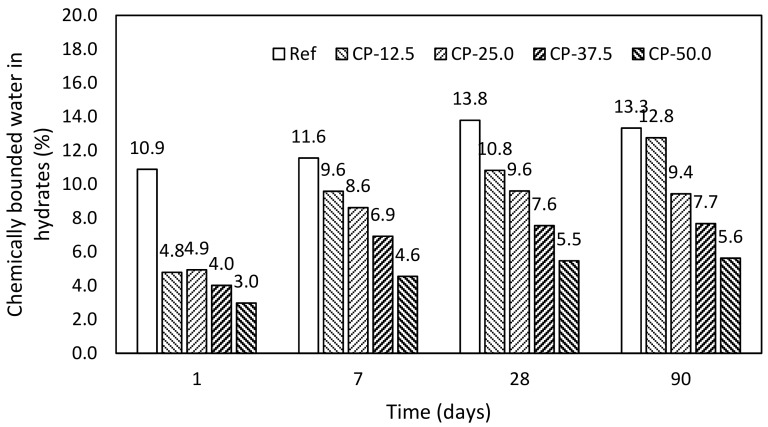
Chemically bounded water in hydrates—pastes with ceramic powder.

Pastes with addition of ceramic powder. Ceramic powder performs an alternative source of active additive to cement-based composites; however, its pozzolanic properties are low in comparison with traditionally used mineral additives, Figure 6. Its reduced activity is visible in the evolution of hydrates in hardened pastes in time. The amount of hydrates after 90 days for the highest applied replacement was nearly similar to that for blast furnace slag after 24 h.

Generally, the experimental results confirmed expected proportions for the studied additives. Obtained values of chemically bounded water in hydrates in studied pastes incorporating various cement supplementary materials were used for the estimation of ultimate values, which perform theoretical amount of hydrates in case of full hydration. Obtained coefficients and ultimate values in terms of Michaelis-Menten equation (Equation (7)) are introduced in Table 4.

Thanks to knowledge of ultimate values of chemically bounded water, it is possible to calculate (Equation (6)) the degree of hydration for given time intervals, thus describing the evolution of hydration in time. Obtained values are shown in Table 5, where the significant lack of this procedure is evident—estimation of ultimate value of binder with prolonged hydration. Determined ultimate values introduce estimation on the basis of relatively short-term measurement with respect to the process of hydration of blended system. Zobal et al. in [16] published results of the long-term properties of Orlik dam concrete, where high level of Portland cement substitution was applied due to the needed hydration heat reduction. Relative increase of mechanical performance after 40 years was 100% for the replacement by 50% of fly ash. However, it is necessary to note that special Portland cement with coarser grading was used for the construction of the studied Orlik dam. The necessity of the correction is obvious in the results shown in Table 5. It is highly unlikely that pastes incorporating fly ash or blast furnace slag completed hydration after 90 days. This is why a correction was applied, taking into the consideration the different kinetics of hydration of blended system.

The correction was partially inspired by the EN 206 [18] implementing *k*-value, which allows the calculation of equivalent mass of the binder *L_eq_*; additionally, the gypsum content was taken into the consideration. The k-values are introduced in Table 3 for traditionally used mineral additives. This approach was also used by Deboucha et al. [36]. On the basis of mineralogical properties of studied ceramic powder, its k-value was estimated to be 0.2 for following work, because of half amount of amorphous phases in comparison to fly ash. The values of corrected degree of hydration are introduced in Table 6; they perform more realistic results with various character of studied mineral additives.

**Figure 7:**
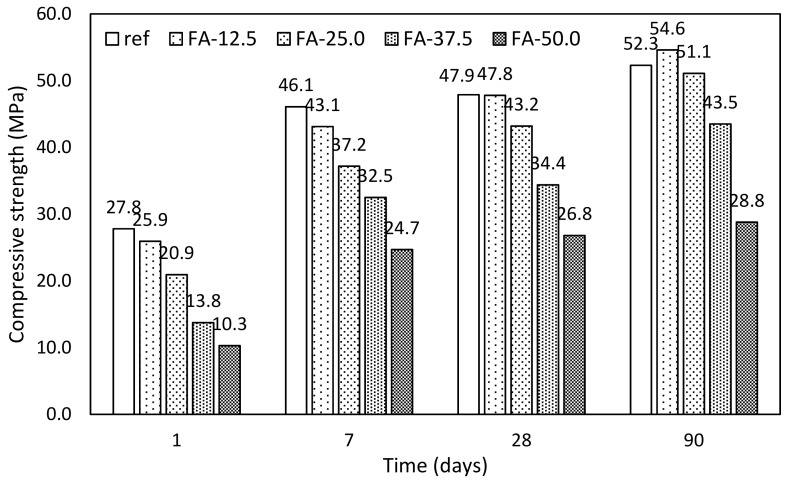
Evolution of compressive strength of mortars with fly ash.

Fly ash. Evolution of mechanical properties in time were studied in terms of compressive strength using mortar samples. Results of mortars with fly ash addition are introduced in Figure 7. These results fairly correspond with the evolution of hydrates determined by thermogravimetry. However, replacement by fly ash up to 25.0% achieves higher mechanical performance after 90 days in comparison with control mixture. 

In particular, compressive strength after 90 days with respect to the control mixture achieved 104, 97.7, 83.2, and 55.1%, respectively, for gradual substitution by fly ash 12.5, 25.0, 37.5, and 50.0% FA. These results correspond with similar experimental works [1,44]. Higher levels of replacement exhibited stronger evolution of mechanical properties in time; however, there was a significant decay with respect to control mixture. Similar results were obtained by Siddique [45,46]. On the other hand, Varga et al. [47] declared that this decay could be supressed by the reduction of water dosage. However, according to Atis [48], application of superplasticizer increased shrinkage of samples.

**Figure 8:**
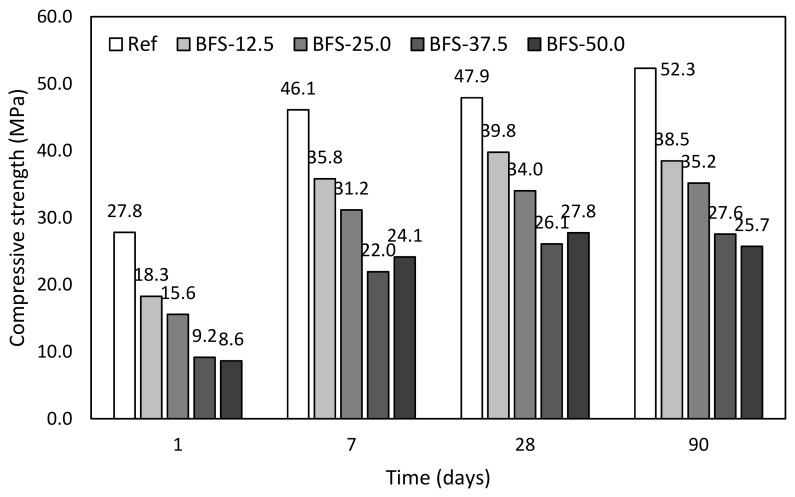
Evolution of compressive strength of mortars with blast furnace slag.

Blast furnace slag. Substitution of OPC by blast furnace slag exhibits lower mechanical performance in comparison with the fly ash (Figure 8). This is probably caused by the filler effect of used FA and more suitable gradation curve of the blend of cement and FA. It is shown by the particle size distribution curves introduced in Figure 2. The filler effect is also powered by the increased porosity with respect to the applied value of w/b. In particular, compressive strength after 90 days with respect to control mixture achieved 73.6, 67.3, 52.8, and 49.1%, respectively, for the gradual substitution by blast furnace slag 12.5, 25.0, 37.5, and 50.0%. However, it is necessary to mention that efficiency of the BFS application is highly dependent on the used w/b ration, dosage of cement and particle size distribution, Figure 2. In addition, blast furnace slag exhibits latent hydraulic properties, so its substitution could be obviously higher in comparison with the fly ash; however, it is necessary to decrease w/b to achieve better mechanical performance. Similar w/b ratio leads to the increased porosity and resulting lower mechanical properties. The physical effect of blast furnace slag is very low, because its particle size distribution is very similar to the cement. Oner and Akyuz in [49] studied blended system to find an optimal dosage of BFS in cement-based composites. They applied various levels of replacement and dosages of mixing water. They concluded positive effect of BFS on mechanical performance for inverse mix formulation in terms of w/b ratio and applied replacement level. This could explain commonly accepted presumption of better strength performance of BFS in comparison to FA, because of application of efficient water reducer and relatively high doses of Portland cement [50], and the fact that BFS exhibits higher efficiency [51]. That also confirms the study by Zhao et al. [52], who applied relatively low dosage of water-reducing admixture; thus, both set of mixtures with BFS and FA reached nearly similar mechanical performance.

**Figure 9:**
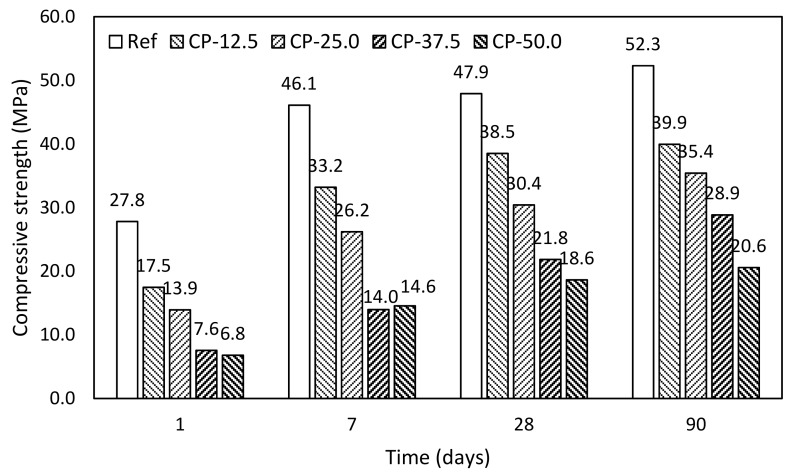
Evolution of compressive strength of mortars with ceramic powder.

Ceramic powder. Mortar samples with ceramic powder reached very similar mechanical performance as those with BFS; however, the highest replacement level exhibited reduced rate of the compressive evolution in time (Figure 9). Compressive strength after 90 days with respect to control mixture achieved 76.3, 67.3, 67.7, and 39.4%, respectively, for gradual substitution 12.5, 25.0, 37.5, and 50.0%. Obtained relative high mechanical performance of mortars with addition of ceramic powder was achieved thanks to the high water adsorption of used mineral additive, which reduces real dosage of mixing and thus porosity of hardened paste. 

Dominant physical effect of ceramic powder in blended systems was confirmed during previous research [53,54]. Pacheco-Torgal and Jalali [39] and Higashiyama [55] studied influence of ceramic powder, and they did not find any harmful effect of this additive in terms of compressive strength; however, control mixture was slightly higher approximately by 10%. Progressive decay of compressive strength obtained corresponds with conclusions of Naceri and Hamina [56], who set optimal replacement level by CP up to 10% by weight. On the other hand, Heikal et al. [57] confirmed increased mechanical performance of SCC incorporating CP and beneficial effect of the application of polycarboxylate-based water reducer.

**Figure 10:**
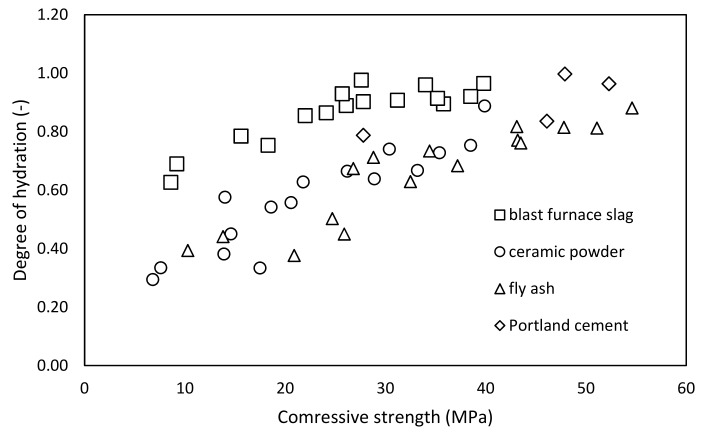
Relation of the degree of hydration and compressive strength.

Generally, mechanical performance of studied mortars with incorporation of various supplementary cementitious materials met the initial expectations. Especially, results of mortars with the ceramic powder highlight the problem of physical effect of mineral additives. Inactive particles of additives are increasing the internal specific surface of hardened paste, which positively stimulates precipitation of the hydrates. This effect is well obvious on the pastes incorporating limestone filler [36,40,42]. That is why the application of an inert mineral additive could cause increased mechanical performance; on the other hand, durability properties expressed often by using performance index [34,58] present more suitable approach for the further incorporation of mineral additives. Combination of the hydration of mineral additive and the physical effect takes place in case of the fly ash and ceramic powder as well. Hence, the study of hydration mechanism in terms of thermogravimetry remains challenging. However, the proposed corrections for the calculation of hydration degree of blended systems exhibit good fit with obtained mechanical performance (Figure 10). 

All data of studied pastes are introduced in Figure 10; nevertheless, lines of particular applied additives are visible. There is evident significant shift of the line for BFS-mixtures hydration degree in comparison with the others used additives after performed corrections. It is probably caused by the estimation of the ultimate amount of chemically bounded water, which was assumed on the basis of 90 days measurements. Present lack of the procedure is mentioned in other works [36,57] in relation to the application of BFS. Nevertheless, proposed procedure for the determination of hydration degree of blended systems reflecting substance of applied mineral additives, partially inspired by k-value concept, exhibits good fit. 

## 4. Conclusions

The performed experimental program was focused on the determination of hydration degree of Portland cement-based mixtures incorporating three various mineral additives: fly ash, blast furnace slag, and ceramic powder. Hydration degree was calculated on the basis of thermogravimetry; however, obtained data were corrected with respect to the activity index of single studied additive. Obtained values were compared with the results of compressive strength determined on the accompanying mortar samples. The principal findings of the paper can be summarized as follows:

- Decomposition of non-aqueous phases in used mineral additive is necessary for inclusion in the calculation of hydration degree;
- The physical effect dominates in the hydration mechanism of ceramic powder. Despite the relatively high mechanical performance of mortar samples, their contribution to hydrate formation is very low;
- The inclusion of an activity index inspired by the k-value in the hydration degree calculation exhibited good fit with the obtained results of compressive strength. The k-value 0.20 was set for ceramic powder with respect to its substance;
- The estimation of the ultimate value of chemically bounded water is crucial for the determination of the hydration degree during time-limited experiments, especially for systems incorporating blast furnace slag.

The estimation of the ultimate reaction capacity of blended binders on the basis of Portland cement is the main task for the future, because the determination of the hydration degree is a supportive tool for achieving a better understanding of the studied binders.

## Figures and Tables

**Table 1:** Chemical composition of used binding components (%) [38].

Component	Ceramic Powder	Blast Furnace Slag	Fly Ash	Portland Cement
SiO_2_	50.7	36.0	52.4	18.5
Al_2_O_3_	20.0	9.0	35.9	6.5
Fe_2_O_3_	6.2	0.3	4.9	2.4
CaO	11.6	43.5	1.2	64.9
MgO	4.8	8.3	0.8	1.0
K_2_O	3.2	0.5	1.4	1.2
Na_2_O	1.3	0.5	-	0.1
TiO_2_	0.8	0.3	2.4	-
SO_3_	1.0	0.5	0.2	4.9

**Table 2:** Phase composition of used additives (%).

Component	Summary Formula	Fly Ash	Blast Furnace Slag	Ceramic Powder
Amorphous portion	-	62.8	83.4	27.1
Quartz	SiO_2_	5.8	-	25.2
Hematite	Fe_2_O_3_	-	-	2.8
Albite	NaAlSi_3_O_8_	-	-	14.0
Microcline	KAlSi_3_O_8_	-	-	7.0
Muscovite	KAl_2_(AlSi_3_O_10_)(OH)_2_	-	-	12.9
Illite	K_0.65_Al_2_(Al_0.65_Si_3.35_O_10_)(OH)_2_	-	-	3.4
Diopside	CaMgSi_2_O_6_	-	-	4.8
Akermanite	Ca_2_MgSi_2_O_7_	-	2.8	2.4
Mullite	Al_6_Si_2_O_13_	31.1	-	-
Anhydrite	CaSO_4_	-	7.5	-
Merwinite	Ca_3_MgSi_2_O_8_	-	5.8	-

**Table 3:** k-values for selected mineral additives [36].

Mineral Additive	k-Value
Blast furnace slag	0.9
Silica fume	2.0
Fly ash (F class)	0.4

**Table 4:** Estimated ultimate content of chemically bounded water using Michealis-Menten equation.

Replacement (%)	Fly Ash		Blast Furnace Slag		Ceramic Powder	
	H_hyd∞_ (%)	K (-)	H_hyd∞_ (%)	K (-)	H_hyd∞_ (%)	K (-)
0	13.14	5.44	13.14	5.44	13.14	5.44
12.0	10.80	22.00	11.55	6.19	12.22	40.78
25.0	10.08	28.20	11.33	4.60	9.73	23.25
37.5	8.40	17.67	10.22	8.62	7.82	22.84
50.0	6.41	21.60	8.68	11.38	5.56	22.11

**Table 5:** The degree of hydration (-) of studied materials in time.

Studied Materials	The Degree of Hydration (-)			
	1 day	7 days	28 days	90 days
Ref.	0.83	0.88	1.05	1.01
FA-12.5	0.51	0.93	0.92	1.00
FA-25.0	0.46	0.84	0.95	1.00
FA-37.5	0.59	0.85	0.99	1.02
FA-50.0	0.58	0.74	1.00	1.05
BSF-12.5	0.80	0.95	1.02	0.98
BSF-25.0	0.84	0.97	1.02	0.97
BSF-37.5	0.74	0.92	0.96	1.05
BSF-50.0	0.68	0.93	0.98	1.00
CP-12.5	0.39	0.79	0.89	1.04
CP-25.0	0.51	0.89	0.99	0.97
CP-37.5	0.51	0.89	0.97	0.98
CP-50.0	0.53	0.82	0.99	1.01

**Table 6:** The degree of hydration (-) of studied materials in time with correction in terms of equivalent binder mass.

Studied Materials	The Degree of Hydration (-)			
	1 day	7 days	28 days	90 days
Ref.	0.79	0.84	1.00	0.96
FA-12.5	0.45	0.82	0.81	0.88
FA-25.0	0.38	0.68	0.77	0.81
FA-37.5	0.44	0.63	0.73	0.76
FA-50.0	0.39	0.50	0.67	0.71
BSF-12.5	0.75	0.90	0.97	0.92
BSF-25.0	0.78	0.91	0.96	0.91
BSF-37.5	0.69	0.85	0.89	0.98
BSF-50.0	0.63	0.86	0.90	0.93
CP-12.5	0.33	0.67	0.75	0.89
CP-25.0	0.38	0.66	0.74	0.73
CP-37.5	0.33	0.58	0.63	0.64
CP-50.0	0.29	0.45	0.54	0.56
